# Rhinovirus Infection and Familial Atopy Predict Persistent Asthma and Sensitisation 7 Years after a First Episode of Acute Bronchiolitis in Infancy

**DOI:** 10.3390/children8100850

**Published:** 2021-09-26

**Authors:** Julie Magnier, Valérie Julian, Aurélien Mulliez, Alexandra Usclade, Emmanuelle Rochette, Bertrand Evrard, Flore Amat, Carole Egron

**Affiliations:** 1 CHU Clermont-Ferrand, Pôle Pédiatrique, Unité d’allergologie de l’enfant, CHU Estaing, 1 Place Lucie et Raymond Aubrac, F-63003 Clermont-Ferrand, France; jmagnier@chu-clermontferrand.fr (J.M.); cegron@chu-clermontferrand.fr (C.E.); 2 Department of Sport Medicine and Functional Explorations, Diet and Musculoskeletal Health Team, CRNH, INRA, University Teaching Hospital of Clermont-Ferrand, University of Clermont Auvergne, F-63003 Clermont-Ferrand, France; vjulian@chu-clermontferrand.fr; 3 CHU Clermont-Ferrand, Unité de Biostatistiques, Direction de la Recherche Clinique et Innovation (DRCI), 58 Rue Montalembert, F-63003 Clermont-Ferrand, France; amulliez@chu-clermontferrand.fr; 4 CHU Clermont-Ferrand, INSERM CIC 1405, F-63003 Clermont-Ferrand, France; ausclade@chu-clermontferrand.fr; 5 CHU Clermont-Ferrand, Service d’Immunologie, CHU Gabriel-Montpied, 58 Rue Montalembert, F63003 Clermont-Ferrand, France; bevrard@chu-clermontferrand.fr; 6 UFR Médecine, Unité ECREIN, Clermont Université, Université d’Auvergne, UMR 1019, UNH, F-63003 Clermont-Ferrand, France; 7 Unité Fonctionnelle de Pneumologie et Allergologie Pédiatrique, Hôpital Robert Debré, 48 Boulevard Sérurier, F-75019 Paris, France; flore.amat@aphp.fr

**Keywords:** acute bronchiolitis, rhinovirus, respiratory syncytial virus, atopy, asthma

## Abstract

Background: We set out to assess the risk factors for asthma outcome in a cohort of infants who experienced their first episode of acute bronchiolitis. Methods: A cohort of 222 infants who were included during a first episode of acute bronchiolitis was prospectively followed. Herein, we present the results of their assessments (symptom history, skin prick tests, specific IgE assay, respiratory function tests) at age seven. Results: Of the 68/222 (30.6%) children assessed at age seven, 15 (22.05%) presented with asthma and were mainly males (*p* = 0.033), 14 (20%) had respiratory allergies, 17 (25%) presented atopic dermatitis and none had a food allergy. Family history of atopy was associated with asthma and sensitisation to aeroallergens at age seven (*p* = 0.003, *p* = 0.007). Rhinovirus (hRV) infection and rhinovirus/respiratory syncytial virus (RSV) co-infection were significantly associated with asthma at age seven (*p* = 0.035, *p* = 0.04), but not with the initial severity of bronchiolitis. Eosinophil counts at ages three and seven were significantly higher in the asthmatics (*p* = 0.01, *p* = 0.046). Conclusion: Any infant, especially male, presenting a first episode of acute bronchiolitis due to hRV with a family history of atopy should be closely monitored via follow-up due to a higher risk for asthma at school age.

## 1. Introduction

Acute bronchiolitis is the most common cause of lower respiratory tract infections in the first year of life. The viruses that are mainly responsible are the respiratory syncytial virus (RSV), which is associated with a more severe disease, and the rhinovirus (hRV), which is associated with more moderate forms of disease but a higher risk of asthma during childhood [1,2]. Most children have a moderate form of the disease, which is usually managed on an outpatient basis. Meanwhile, hospitalisation is required for severe forms of the disease [3,4].

Asthma is a common disease in the paediatric population, with a prevalence of approximately 8.3% among children aged 0–17 years in the USA [5], and 11% in France [6]. The prevalence decreases with age, with a higher rate of recurrent wheezing in the younger age groups [7].

A few prospective studies have been conducted on asthma after acute bronchiolitis, with the type of virus involved and familial atopic status appearing as determinant factors. In the available cohorts, RSV is associated with severe initial infection and viral recurrent wheezing in the first years of life, whereas hRV is associated with long term asthma genesis in an atopic context. However, the available cohorts were conducted specifically in severe cases, notably in hospitalized cases [7,8]. To our knowledge, no prospective study has been conducted to date focusing on an acute bronchiolitis population, regardless of its severity, including mild forms managed at home [7]. Predicting the risk of asthma later in life in infants is difficult [9]. Thus, using a phenotype approach for the early identification of children at risk for asthma in the long-term is crucial.

We prospectively followed a cohort of infants aged under one year with a first episode of acute bronchiolitis, either hospitalised or cared for at home [10]. Herein, we present the results of their assessments at age seven. The aim of our prospective cohort study of infants with acute bronchiolitis was to evaluate the roles of early viral infections (particularly the types of viruses involved and the severity of the infection), as well as the overall aeroallergen sensitisations and their associations with asthma at age seven.

## 2. Materials and Methods

### 2.1. Study Design

A cohort of infants aged under one year consulting for their first episode of acute bronchiolitis during the 2011–2012 winter epidemic season at the University Hospital of Clermont-Ferrand (Clermont-Ferrand, Rhône-Alpes Auvergne, France) was prospectively re-assessed at age seven in 2019.

### 2.2. Ethics

All the children and their parents gave written informed consent for their participation and follow-up at three and seven years old. The research protocol for this study was previously approved by our institutional review board (*Comité de Protection des Personnes Nord Ouest IV*, France, Approval No. 2018-A02659-46).

### 2.3. Study Population

All children aged under one year presenting at the Paediatric Emergency Department for a first episode of acute bronchiolitis were considered for inclusion, from the mild forms managed in primary care to the most severe forms admitted to an intensive care unit. Infants with bronchopulmonary dysplasia, history of prematurity under 34 weeks, cystic fibrosis, known immune deficiencies, suspected primary ciliary dyskinesia, congenital heart disease or acute renal failure were excluded from the study. At the inclusion, the most common pathogen was RSV, which was found in 166 samples (76%). Meanwhile, HRVs were found in 72 of the samples (33%). Co-infection with RSV and HRV was detected in 41 patients (19%) [10]. We first re-evaluated the children three years after inclusion. At this point in the follow-up, 46.8% presented with recurrent bronchial obstruction [11]. Only a family history of atopy was associated with asthma, and not the type of virus involved or the severity of acute bronchiolitis episode. RSV-hRV co-infection was associated with aeroallergen sensitisation.

The variables recorded at inclusion have been described elsewhere [10]. Briefly, demographic data were first obtained from the parents using a structured questionnaire. Acute bronchiolitis was defined as mild, moderate or severe according to a severity score calculated from SpO_2_, respiratory rate and respiratory effort on admission (Wainwright score [12]). The infants were hospitalised according to the French Guidelines (HAS 2019 [13]). Hospitalisation was required for young infants aged under six weeks and/or experiencing respiratory distress, major deterioration in general condition, risk of clinical dehydration or compromised home care (familial socio-economic context or limited use of care due to geographic location of the domicile). A nasopharyngeal aspirate was collected either in the emergency room or within the first 24 h following admission to detect respiratory viruses [10]. A telephone survey was also conducted. The parents of each child were contacted every three months until age three. A standardised questionnaire designed to elicit information on respiratory symptoms was used at every interview.

At age three, a complete medical check-up with an immunological profile was performed at the Outpatients Department of CHU-Estaing Hospital [11]. Familial atopy history was considered in cases of personal atopy history (eczema, rhinoconjunctivitis, food anaphylaxis or asthma) in one parent, sister or brother. At age seven, the parents of each child assessed at age three were contacted for a new assessment. The patient was declared lost to follow-up after three fruitless phone calls.

### 2.4. Clinical Data

At age seven, a clinical questionnaire was administered to assess data on respiratory symptoms (day or night cough, discomfort during exercise, use of salbutamol), respiratory treatments (corticosteroid p.o. or inhaled, long-acting bronchodilator), hospitalisation for asthma, wheezing outside viral episodes, personal atopy (presence of atopic dermatitis, eczema, rhinoconjunctivitis, food anaphylaxis from birth), environmental exposure to tobacco smoke (currently or during pregnancy), pets, type of housing, day-care attendance and number of siblings. Sociodemographic data were also collected, including sex, weight, height, and assessment of precariousness using the EPICES (Assessment of Precariousness and Health Inequalities in Health Examination Centers) questionnaire [14]. According to the French Health Authority, ‘precariousness’ is defined as a state of social instability characterised by the loss of one or more of those securities, particularly employment, that enable individuals and families to assume their professional, family and social responsibilities and enjoy their fundamental rights. Precariousness can thus manifest itself in several areas, such as income, housing, employment, educational qualifications, social protection, leisure, culture and health. A score of over 30 was taken as indicative of precariousness. Physical examination data, including symptoms of asthma, existence of eczema or signs of rhinoconjunctivitis and chest deformity were also collected.

### 2.5. Pulmonary Function Testing

Spirometry was performed using the Jaeger MasterScreeen. The spirometer was calibrated each morning using a 3-litre syringe. A nose clip was applied, and the children underwent a forced vital capacity (FVC) test in accordance with the American Thoracic Society/European Thoracic Society 2005 spirometry standards [15]. For each child, we set out to obtain three FVC manoeuvres that meet the ATS/ERS acceptability criteria. The forced expiratory volume in 1 s (FEV1), forced vital capacity (FVC), peak expiratory flow (PEF), maximum mid-expiratory flow (MMEF), forced expiratory flow (FEF), maximum expiratory flow (MEF) and maximum expiratory flow at 25%, 50% and 75% expiratory volumes (MEF 25, MEF 50, MEF 75) were measured.

Interrupter resistance (Rint) was measured using a Spiro Dyn apparatus (Spiro Dyn’R, Toulouse) calibrated daily for flow. Rint was measured during a 100-ms occlusion during expiration. While the measurements were carried out, each child was seated and asked to breathe quietly, connected to the flowmeter by a mouthpiece. Measurements were made with the child’s head held in the neutral position, with the cheeks and chin held by the physician. The examinations were performed with and without a bronchodilator. For bronchodilator response, 400 µg of salbutamol was inhaled through the spacer device. Fifteen minutes after the administration of the drug, each child underwent further spirometry to evaluate the bronchodilator response.

Normal pattern was defined by normal FVC and normal FEV_1_/FVC ratios. Significant obstructive airflow disorder was defined by FEV_1_/FVC < 0.8. We considered significant reversibility for an increase of 12% in FEV_1_ or a decrease of 35% in Rint [16].

### 2.6. Biological Assessment

Prick tests were carried out by the same trained clinical research nurse to optimise reproducibility. Skin prick tests included histamine and isotonic saline as controls, main aeroallergens (*Dermatophagoides pteronyssinus*, *Dermatophagoides farinae*, *Alternaria*, timothy grass, birch, cats, dogs) and main trophallergens (egg, peanut, walnut, almond, hazelnut, soy). Papule diameter was measured at 20 min. The prick test was considered positive with a diameter equal to or larger than 3 mm compared with the control. Sensitisations to aeroallergens and trophallergens were confirmed by a unitary assay of specific serum IgE by ImmunoCAP (Phadia 250^®^, Phadia AB, Uppsala, Sweden). ImmunoCap Phadia 250^®^ provides a quantitative result ranging from 0.1 to 100 kUA/L. We took 0.35 kUA/L as the positive threshold. Multi-sensitisation was defined if the tests (SPT or IgE assay) were positive for at least two allergens, except for co-sensitisation to *Dermatophagoides pteronyssinus* and *Dermatophagoides farinae*, which was considered as monosensitisation. A blood test was performed to assess eosinophilia, defined for levels up to 0.300 G/L [17].

### 2.7. Asthma Outcome

Asthma was defined according to the frequency of the following symptoms: asthma symptoms (tachypnea, wheezing, expiratory stridor, respiratory chest retraction), and doctor-diagnosed wheezes or use of anti-asthmatic medication with a medical prescription. If ≥3 respiratory symptoms were documented ≥2 times or if any such episode lasted ≥4 weeks, then the subject was classified as having asthma [18,19].

### 2.8. Statistical Analysis

Statistics were computed using Stata v15 (StataCorp, College Station, TX, USA). The study sample was described by frequency and associated percentage for categorical data and by mean ± standard deviation, by deviation when the data were normally distributed or by median and interquartile range (IQR) for continuous data. Univariate analysis of asthma was conducted using the chi-squared test (or Fisher’s exact test when appropriate) for categorical data and using Student’s *t*-test (or the Mann–Whitney test when data were non-normal) for continuous data. Results are shown as odds ratio with 95% confidence interval. The same methods were used to analyse associations between the development of allergic sensitisation and the patient’s characteristics, as well as for asthma analysis in the subgroup of patients with aeroallergen sensitisation. All the tests were two-sided, and a *p*-value < 0.05 was considered significant.

## 3. Results

Overall, 222 children were initially recruited (2011–2012). Of this number, 154/222 (69.4%) were assessed at age three in 2014–2015. Finally, 68/222 (30.6%) completed the entire follow-up period at age seven in 2019.

The children who were lost to follow-up (Table 1) did not differ significantly from those assessed at age seven in terms of sex, gestational age at birth, family history of asthma, passive tobacco smoking, initial severity of bronchiolitis and initial care for acute bronchiolitis (hospitalisation or ambulatory care).

### 3.1. Description of the Population

The population of children assessed at age seven comprised mainly males (43/68; 63.2%) aged 7.68 ± 0.3 years. Median body mass index was 15.6 kg/m^2^ (±2.8 SD). With regards to acute bronchiolitis history at inclusion, four patients (4/68; 5.8%) presented with severe acute bronchiolitis and were hospitalised at an intensive care unit. The others were mainly cared for in the hospital (53 children/68, 77.9%). The viruses involved were RSV in 76.4%, hRV in 23.5% and RSV-hRV co-infection in 10.3% of the cases. Twenty-six children (26/68; 38.2%) were currently exposed to tobacco smoking and 10/68 (14.7%) during pregnancy. Fifty-six children (56/68; 82.3%) lived in houses and 12/68 (17.7%) in apartments. Animals were present at home for 48/68 (70.6%) of the subjects and cats for 25/68 of them (36.8%). Precariousness was suspected based on the EPICES score of seven patients (7/68; 10.3%).

### 3.2. Asthma

We found an asthma rate of 22.05% in our population (*n* = 15/68 children). Twelve (12/15; 80%) have presented asthma since age three. Three (3/15; 20%) developed asthma at school age. Four children (4/68; 5.9%) had already been hospitalised for an asthma attack, 10 (10/68; 14.7%) had wheezing episodes outside of a viral episode, 32 (32/68; 47%) received occasional treatments with salbutamol and 16 (16/68; 23.5%) took daily background therapy. Independent of the validated asthma diagnosis, several children had already received anti-asthmatic treatment; one of them was not asthmatic at age seven. Regarding respiratory functional exploration, none of the children had any obstructive ventilation disorder (FEV_1_ 104% ± 9%, FEV_1_/FVC 107% ± 6 (mean ± SD)). We noted significant reversibility in 16 children (16/68; 23.5% of our population), having observed it in five asthmatic patients (5/15; 33%) and in 11 non-asthmatics (11/53; 21%).

### 3.3. Allergy and Sensitisation

We noted an atopic family history for 47 patients (47/68; 69.1%). Seventeen patients (17/68; 25%) presented atopic dermatitis history, 14 (14/68; 20.1%) allergic respiratory symptoms and none had a food allergy.

Seven of the asthmatic patients were sensitised to an aeroallergen (*p* = 0.009) and none to a trophallergen. With regards to aeroallergens, we highlighted a sensitisation to mites in 10 children (10/68; 14.7%), to *Alternaria alternata* in two (2/68; 2.9%), to grasses in seven (7/68; 10.3%), to timothy grasses in five (5/68; 7.3%) and to cats in five (5/68; 7.3%) (Table 2).

A higher eosinophil count was observed in active asthmatics aged seven, evaluated at ages three and seven (Table 3). Eosinophilia considered as an eosinophil count with ≥300 cells μL^−1^ did not differ, evaluated at ages three (*p* = 0.361) and seven (*p* = 0.178).

### 3.4. Risk Factors for Asthma and Sensitisation in the Long Term

In the univariate analysis, being male, having a family history of allergy, rhinovirus infection or rhinovirus-RSV acute bronchiolitis and greater blood eosinophilia assessed at ages three and seven were associated with asthma at age seven (Table 3). The initial severity of acute bronchiolitis was not associated with asthma at age seven using the Wainwright score (*p* = 0.457), the hospitalisation variable (*p* = 0.73) or the intensive care requirement (*p* = 0.57). Exposure to tobacco smoke (currently or during pregnancy), precariousness or the presence of a cat at home during infancy was not associated with asthma at age seven.

Only family history of atopy seemed to be associated with the development of allergic sensitisation (*p* = 0.017). Initial infections with hRV or RSV-hRV were not significantly associated with sensitisation at age seven (*p* = 0.506 and *p* = 0.345, respectively). Notably, exposure to tobacco smoke (currently or during pregnancy) was not linked with the development of sensitisation at age seven (*p* = 0.604 and *p* = 0.047).

## 4. Discussion

In this prospective cohort study of infants presenting a first episode of acute bronchiolitis before one year old, we sought to extend our previous findings on the roles of early viral infections, as well as overall aeroallergen sensitisations and their associations with asthma at age seven. This is the first prospective study that included mild to severe cases, care at home or hospitalised, and with a long-term follow up.

### 4.1. Asthma Development

We found a asthma development rate of 22%, which is comparable to the few other cohorts available of infants hospitalised for acute bronchiolitis (15–34%) [8,19,20]. Most of the patients presented normal respiratory function, but with airway hyperresponsiveness (AHR) without active symptomatic asthma. This could be the consequence of remodelling and structural changes in connection with their previous symptomatic asthma [21]. These patients were not considered asthmatic in the absence of symptoms.

### 4.2. Asthma and Risk Factors

The main factors predicting asthma development for these children seemed to be male sex, family history of allergy and rhinovirus infection, isolated or in association with RSV. Moreover, the risk of sensitisation to aeroallergen at age seven seemed to be correlated only with family history of atopy.

Notably, the male sex had already been reported as a risk factor for wheezing [22].

Rather than acute bronchiolitis severity, the causal virus involved seems to play a main role in asthma genesis and its phenotype in the long term with two distinct trajectories. At preschool age, the predominant asthma phenotype is the viral recurrent wheezer, with a significant decrease in prevalence at school age and a low risk of chronic obstructive pulmonary disease (COPD) in adulthood [23,24]. As several studies have already shown [21,23,24,25,26], RSV induces more severe initial infections and is involved in the genesis of viral recurrent wheezers, which become minority phenotypes beyond five years old. In our study which evaluated children at school age, acute bronchiolitis due to rhinovirus infection (associated or not with RSV) was associated with active asthma at age seven. Although the precise mechanism is still unclear, the impact of hRV seems to be linked to family atopy status. Both allergic sensitisation and rhinovirus-induced wheezing illnesses in early life increase asthma risk in the long term. Allergic sensitisation could result in a decreased innate immune response to hRV that could generate greater degrees of airway inflammation and the potential for airway remodelling and loss of lung function over time [27,28]. Our results are consistent with atopic status (represented by familial atopic history) and hRV infection having major roles in long-term asthma genesis. In addition, the first hRV acute bronchiolitis in an atopic infant should be considered as a predictive factor of persistent atopic asthma at school age, as represented by mild allergic asthma and severe asthma with multiple allergic comorbidities [29,30]. These infants require the specific attention of paediatricians in the long term due to the risk of COPD development for those phenotypes.

### 4.3. Allergy and Sensitisation

The importance of parental atopy has been shown previously [31]. Having one atopic parent leads to atopic disease development in 20–40% of children, which rises to 40–60% if both parents are atopic. Independent of the virus involved, family history of atopy appears to be the main risk factor for the development of asthma or personal atopy [32]. This is one of the main criteria in many risk algorithms for asthma development [33,34]. In our subjects, all of the sensitised children had a family history of atopy.

### 4.4. Type 2 Inflammation Marker

Early eosinophilia stands out as a potential risk factor for persistent asthma. T2 eosinophilic inflammation is most commonly associated with atopy and allergic asthma [35,36]. Eosinophils release proinflammatory cytokines, leukotrienes [30] and specific basic proteins that are responsible for the damage inflicted on the bronchial epithelium. Several studies suggest that an eosinophil count ≥ 300 cells µL^−1^ in young children could be considered a risk factor [37,38]. Eosinophilia seems to predict wheezing at school age and is correlated with asthma severity, including exacerbation severity and corticosteroid response [32,36,39,40]. In our study, eosinophils were significantly higher in the asthmatic population at each evaluation (age three, age seven). The cut-off of the count ≥ 300 cells µL^−1^ was not significant, possibly owing to our restricted asthmatic population. However, all of these observations argue for an early eosinophil assay to identify infants at risk of persistent and severe asthma. The environment plays a primordial role in asthma genesis, particularly tobacco smoking [25,28]. Contrary to literature data, environmental exposure represented by post-natal passive tobacco smoking, in utero passive tobacco smoking, cat exposure and precariousness were not associated with asthma at age seven in our study. The sample size of our cohort that completed the follow-up is too limited to evaluate their impacts on asthma genesis [23,26,29].

### 4.5. Study Limitations

Finally, the size of our population is clearly the main limitation of our study. Many infants were lost to follow-up during the study. The consequences are not negligible for our results, with a lack of power and a possible selection bias to clearly identify the risk factors for asthma development in the long term (especially eosinophilia or tobacco smoking exposure, as specified previously). Likewise, multivariate analysis could not be performed to confirm univariate results and could be a source of confounding bias. Nevertheless, our results are in line with previous cohort studies and provide additional long-term data pertaining to children with acute bronchiolitis during infancy, including those without atopic familial history and with mild to moderate forms of disease. A case control study with children who did not have acute bronchiolitis during infancy could precisely determine the risk factors for asthma development in the long term after an episode of acute bronchiolitis.

## 5. Conclusions

Precise pneumological assessments should be conducted to identify infants who are at higher risk of asthma in the long term during a first episode of acute bronchiolitis, regardless of its severity. The aim of early phenotyping with virology and atopic status assessment is to identify at an early stage infants who are at risk for asthma development in the long term, because of the COPD risk present in asthma phenotypes active after school age. Thus, paediatricians should offer to those patients personalised prevention, follow-up and treatment.

Any infant, especially male, presenting a first episode of acute bronchiolitis due to hRV with a family history of atopy should be closely monitored via follow-up because of a higher risk for asthma at school age and the potential sequelae in adulthood with COPD development.

## Figures and Tables

**Table 1:** Characteristics of the population: children lost to follow-up/children assessed at age 7.

*n* (%)	Lost to Follow-Up *N* = 154 (69)	Children Assessed at Age 7 *N* = 68 (31)	*p*-Value
Male, *n* (%)	88 (57.14)	43 (63.24)	0.395
Prematurity	18 (11.69)	10 (14.71)	0.532
Caesarean	14 (20.9)	8 (14.04)	0.319
Hospitalisation	119 (77.27)	53 (77.94)	0.912
Reanimation	7 (4.55)	4 (5.88)	0.740
Ventilator assistance	58 (37.66)	28 (41.18)	0.620
Familial asthma	40 (32.79)	25 (37.31)	0.531
Tobacco smoking in pregnancy *n* (%)	18 (26.87)	8 (13.11)	0.053

**Table 2:** Aeroallergen sensitisation profiles.

	Asthma *N* = 15	No Asthma *N* = 53	*p*-Value
Aeroallergen sensitisation *n* (%)	8/15 (53)	8/53 (15)	0.009
Mites *n* (%)	4 (27)	6 (11)	0.21
*Alternaria alternata n* (%)	1 (7)	1 (2)	0.395
Grasses *n* (%)	3 (20)	4 (8)	0.18
Timothy *n* (%)	2 (13)	3 (6)	0.303
Cats *n* (%)	2(13)	3 (6)	0.303

**Table 3:** Factors associated with asthma at age 7.

	Asthma *N* = 15	No Asthma *N* = 53	*p*-Value
Sex			
*Male, n* (%)	13 (86.67)	30 (56.6)	0.033
Causal virus at inclusion			
*hRSV n* (%)	11(73)	41 (79)	0.73
*hRV n* (%)	7 (47)	9 (17)	0.035
*hRSV-hRV n* (%)	4 (27)	3 (6)	0.04
Family allergy history *n* (%)	15 (100)	32 (61)	0.003
Personal atopic history			
*Rhinitis n* (%)	7 (47)	4 (8)	0.001
*Respiratory allergy n* (%)	7 (47)	13 (24)	0.009
*Eosinophils at 3 years (G/L mean [95% CI])*	0.239 [0.178; 0.350]	0.163 [0.091; 0.212]	0.01
*Eosinophils at 7 years (G/L mean [95% CI])*	0.490 [0.180; 0.800]	0.255 [0.130; 0.515]	0.046
Environmental exposures			
*Tobacco smoking during pregnancy n* (%)	4 (27)	6 (11)	0.21
*Passive tobacco smoking n* (%)	5 (33)	30 (56.6)	0.658
*Cat n* (%)	5 (33)	20 (38)	0.755

## Data Availability

The datasets used and/or analysed during the current study are available from the corresponding author upon reasonable request.
